# Language of fungi derived from their electrical spiking activity

**DOI:** 10.1098/rsos.211926

**Published:** 2022-04-06

**Authors:** Andrew Adamatzky

**Affiliations:** Unconventional Computing Laboratory, UWE, Bristol, UK

**Keywords:** fungi, electrical activity, action potential, language

## Abstract

Fungi exhibit oscillations of extracellular electrical potential recorded via differential electrodes inserted into a substrate colonized by mycelium or directly into sporocarps. We analysed electrical activity of ghost fungi (*Omphalotus nidiformis*), Enoki fungi (*Flammulina velutipes*), split gill fungi (*Schizophyllum commune*) and caterpillar fungi (*Cordyceps militaris*). The spiking characteristics are species specific: a spike duration varies from 1 to 21 h and an amplitude from 0.03 to 2.1 mV. We found that spikes are often clustered into trains. Assuming that spikes of electrical activity are used by fungi to communicate and process information in mycelium networks, we group spikes into words and provide a linguistic and information complexity analysis of the fungal spiking activity. We demonstrate that distributions of fungal word lengths match that of human languages. We also construct algorithmic and Liz-Zempel complexity hierarchies of fungal sentences and show that species *S. commune* generate the most complex sentences.

## 1. Introduction

Spikes of electrical potential are typically considered to be key attributes of neurons, and neuronal spiking activity is interpreted as a language of a nervous system [1–3]. However, almost all creatures without nervous system produce spikes of electrical potential—Protozoa [4–6], Hydrozoa [7], slime moulds [8,9] and plants [10–12]. Fungi also exhibit trains of action-potential-like spikes, detectable by intracellular and extracellular recordings [13–15]. In experiments with recording of electrical potential of oyster fungi *Pleurotus djamor*, we discovered two types of spiking activity: high-frequency (period 2.6 min) and low-frequency (period 14 min) [13]. While studying another species of fungus, *Ganoderma resinaceum*, we found that the most common width of an electrical potential spike is 5–8 min [16]. In both species of fungi, we observed bursts of spiking in the trains of the spike similar to that observed in the central nervous system [17,18]. While the similarity could be just phenomenological, this indicates a possibility that mycelium networks transform information via interaction of spikes and trains of spikes in manner homologous to neurons. First evidence has been obtained that indeed fungi respond to mechanical, chemical and optical stimulation by changing pattern of its electrically activity and, in many cases, modifying characteristics of their spike trains [19,20]. There is also evidence of electrical current participation in the interactions between mycelium and plant roots during formation of mycorrhiza [21]. In [22], we compared complexity measures of the fungal spiking train and sample text in European languages and found that the ‘fungal language’ exceeds the European languages in morphological complexity.

In our venture to decode the language of fungi, we first uncover if all species of fungi exhibit similar characteristics of electrical spiking activity. Then we characterize the proposed language of fungi by distributions of word length and complexity of sentences.

There is an emerging body of studies on language of creatures without a nervous system and invertebrates. Biocommunication in ciliates [23] include intracellular signalling, chemotaxis as expression of communication, signals for vesicle trafficking, hormonal communication and pheromones. Plants communication processes are seen as primarily sign-mediated interactions and not simply an exchange of information [24,25]. Evidences of different kinds of chemical ‘words’ in plants are discussed in [26,27]. Moreover, a modified conception of language of plants is considered to be a pathway towards ‘the de-objectification of plants and the recognition of their subjectivity and inherent worth and dignity’ [28]. A field of the language of insects has been developed by Karl von Frisch and resulted in his Nobel Prize for detection and investigation of bee languages and dialects [29,30]. An issue of the language of ants, and how species hosted by ants can communicate the ants language, was firstly promoted in 1971 [31]. In the early 1980s, analysis of the ants’ language using information theory approaches was proposed [32]. The approach largely succeeded in analysis of ants’ cognitive capacities [33–36].

We recorded and analysed, as detailed in §2, electrical activity of ghost fungi (*Omphalotus nidiformis*), Enoki fungi (*Flammulina velutipes*), split gill fungi (*Schizophyllum commune*) and caterpillar fungi (*Cordyceps militaris*). The phenomenological characteristic of the spiking behaviour discovered are presented in §3. Linguistic analysis and information and algorithmic complexity estimates of the spiking patterns are given in §4.

## 2. Experimental laboratory methods and analysis

Four species of fungi have been used in experiments: *Omphalotus nidiformis* and *Flammulina velutipes*, supplied by Mycelia NV, Belgium (mycelium.be), *Schizophyllum commune*, collected near Chew Valley lake, Somerset, UK (approximate coordinates 51.34949164156282,−2.622511962302647), *Cordyceps militaris*, supplied by Kaizen Cordyceps, UK (kaizencordyceps.co.uk).

**Figure 1.**
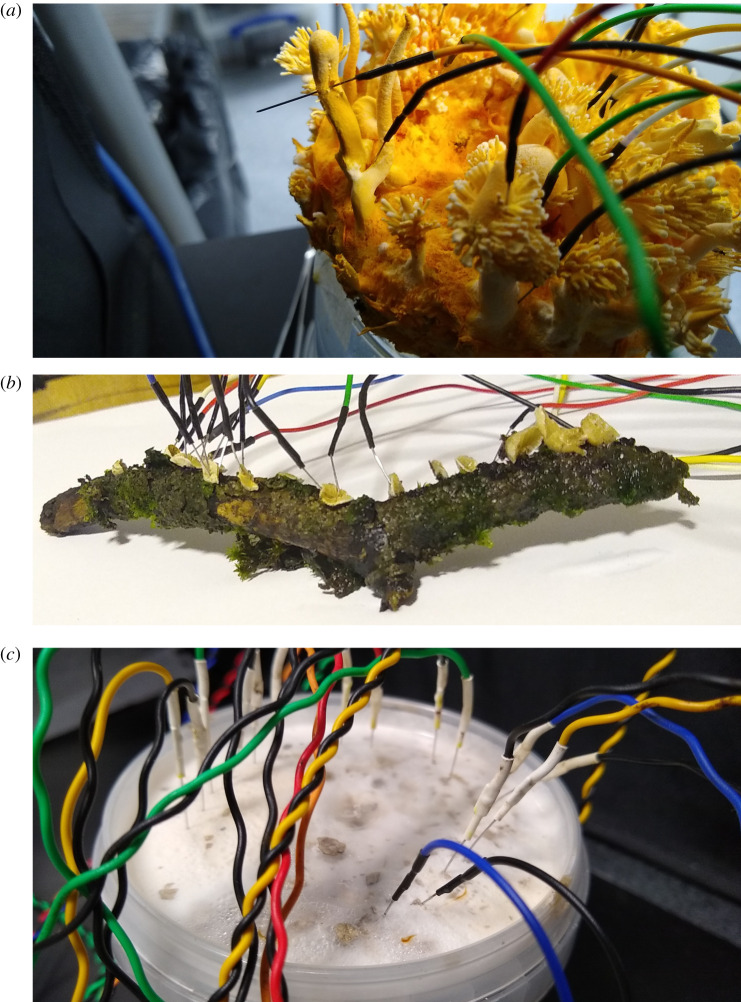
Photographs of pairs of differential electrodes inserted in (*a*) *C. militaris*, the block of a substrate colonized by the fungi was removed from the plastic container to make a photo after the experiments, (*b*) *S. commune*, the twig with the fungi was removed from the humid plastic container to make a photo after the experiment, (*c*) *F. velutipes*, the container was kept sealed and electrodes pierced through the lid.

Electrical activity of the fungi was recorded using pairs of iridium-coated stainless steel sub-dermal needle electrodes (Spes Medica S.r.l., Italy), with twisted cables and ADC-24 (Pico Technology, UK) high-resolution data logger with a 24-bit A/D converter, galvanic isolation and software-selectable sample rates all contributing to a superior noise-free resolution. Each pair of electrodes reported a potential difference between the electrodes. The pairs of electrodes were pierced into the substrates colonized by fungi or, as in the case of *S. commune*, in the sporocarps, as shown in figure 1. Distance between electrodes was 1–2 cm. We recorded electrical activity one sample per second. We recorded eight electrode pairs simultaneously. During the recording, the logger has been doing as many measurements as possible (typically up to 600 per second) and saving the average value. The acquisition voltage range was 78 mV. *Schizophyllum commune* has been recorded for 1.5 days, other species for *ca* 5 days. The experiments took place at temperature 21°C, *ca* 80% humidity, in darkness.

**Figure 2.**
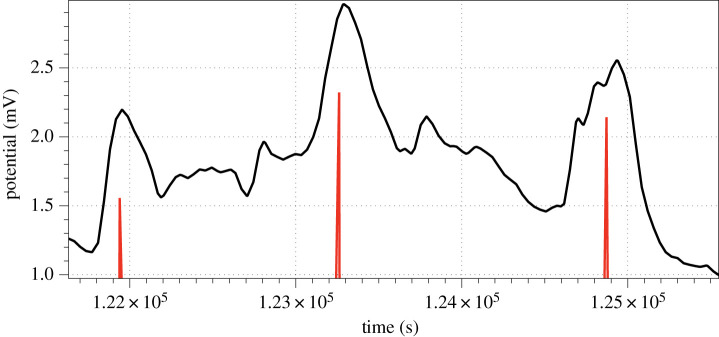
Example of spike detection. Temporal position of each spike is shown by red vertical line. The minor shift of the vertical lines away from the summits is consistent all over the recording and therefore does not affect the results of the analysis.

Spikes of electrical potential have been detected in a semi-automatic mode as follows. For each sample measurement *x*_*i*_, we calculated average value of its neighbourhood as $a_{i}={\left(4\cdot w\right)}^{-1}\cdot \sum_{i-2\cdot w\leq j\leq i+2\cdot w}x_{j}$. The index *i* is considered a peak of the local spike if |*x*_*i*_| − |*a*_*i*_| > *δ*. The list of spikes were further filtered by removing false spikes located at a distance *d* from a given spike. Parameters were species specific, for *C. militaris* and *F. velutipes*, *w* = 200, *δ* = 0.1, *d* = 300; for *S. commune,* *w* = 100, *δ* = 0.005, *d* = 100; for *O. nidiformis,* *w* = 50, *δ* = 0.003, *d* = 100. An example of the spikes detected is shown in figure 2. Over 80% of spikes have been detected by such a technique.

## 3. Characterization of the electrical spiking of fungi

**Figure 3.**
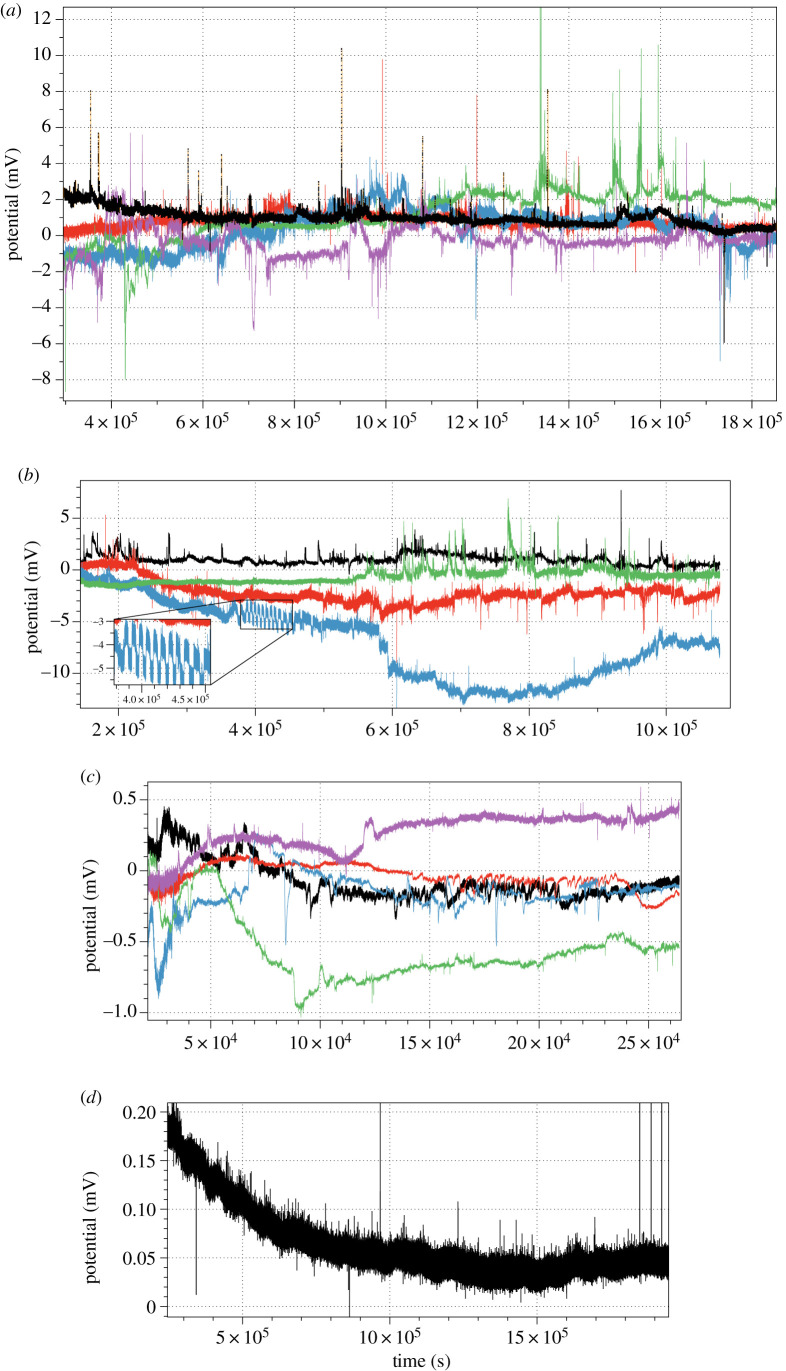
Examples of electrical activity of (*a*) *C. militaris*, (*b*) *F. velutipes*, insert shows zoomed in burst of high-frequency spiking, (*c*) *S. commune* and (*d*) *O. nidiformis*. Colours reflect recordings from different channels.

**Figure 4.**
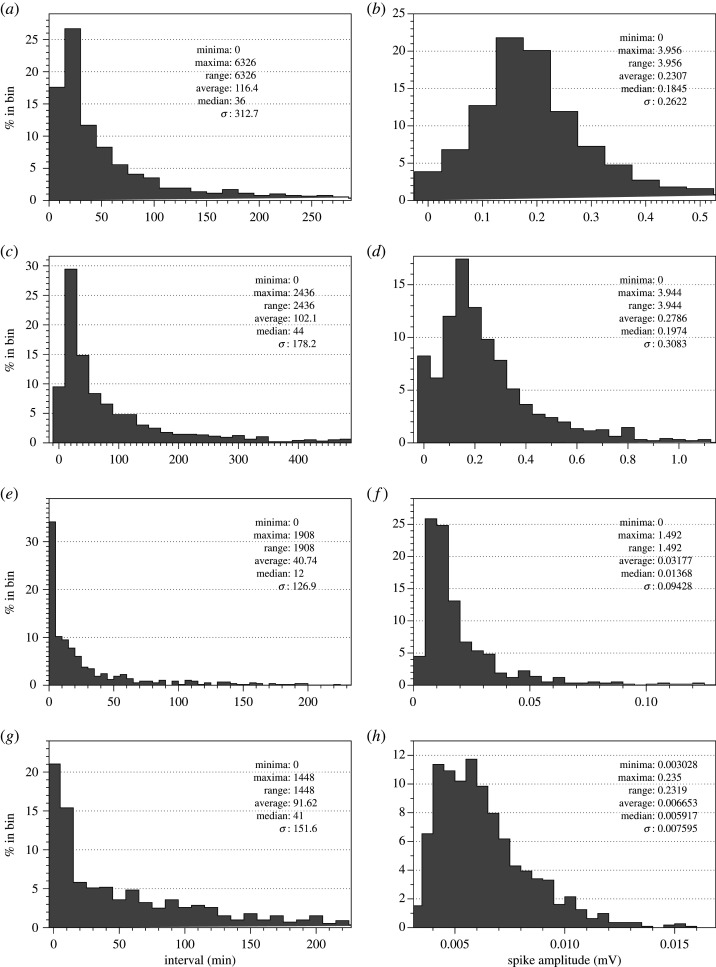
Distribution of intervals between spikes (*a*,*c*,*e*,*g*) and average spike amplitude (*b*,*d*,*f*,*h*) of (*a*,*b*) *C. militaris*, (*c*,*d*) *F. velutipes*, (*e*,*f*) *S. commune* and (*g*,*h*) *O. nidiformis*.

Examples of electrical activity recorded are shown in figure 3. Intervals between the spikes and amplitudes of spikes are characterized in figure 4 and table 1.

**Table 1.** Characteristics of electrical potential spiking: number of spikes recorded, average interval between spikes and average amplitude of a spike.

species	no. spikes	interval (min)	amplitude (mV)
*C. militaris*	881	116	0.2
*F. velutipes*	958	102	0.3
*S. commune*	530	41	0.03
*O. nidiformis*	1117	92	0.007

*Cordyceps militaris* shows the lowest average spiking frequency among the species recorded (figures 3*a* and 4*a*): average interval between spikes is nearly 2 h. The diversity of the frequencies recorded is highest among the species studied: standard deviation is over 5 h. The spikes detected in *C. militaris* and *F. velutipes* have highest amplitudes: 0.2 and 0.3 mV, respectively. Variability of the amplitudes in both species is high, standard deviation nearly 0.3.

Enoki fungi *F. velutipes* show a rich spectrum of diverse patterns of electrical activity which combines low- and high-frequency oscillations (figure 3*b*). Most commonly exhibited patterns are characterized by low-frequency irregular oscillations: average amplitude 0.3 mV (figure 4*d*) and average interval between two spikes is just over 1.5 h (figure 4*c* and table 1). There are also bursts of spiking showing a transition from a low-frequency spiking to high frequency and back, see recording in blue in figure 3*b*. There are 12 spikes in the train, average amplitude is 2.1 mV, *σ* = 0.1, average duration of a spike is 64 min, *σ* = 1.7.

*Omphalotus nidiformis* also show low amplitude and low-frequency electrical spiking activity with the variability of the characteristics highest among species recorded (figure 3*a* and table 1). Average interval between the spikes is just over 1.5 h with nearly 2.5 h standard variation (figure 4*g*). Average amplitude is 0.007 mV but the variability of the amplitudes is very high: *σ* = 0.006 (figure 4*h*).

**Figure 5.**
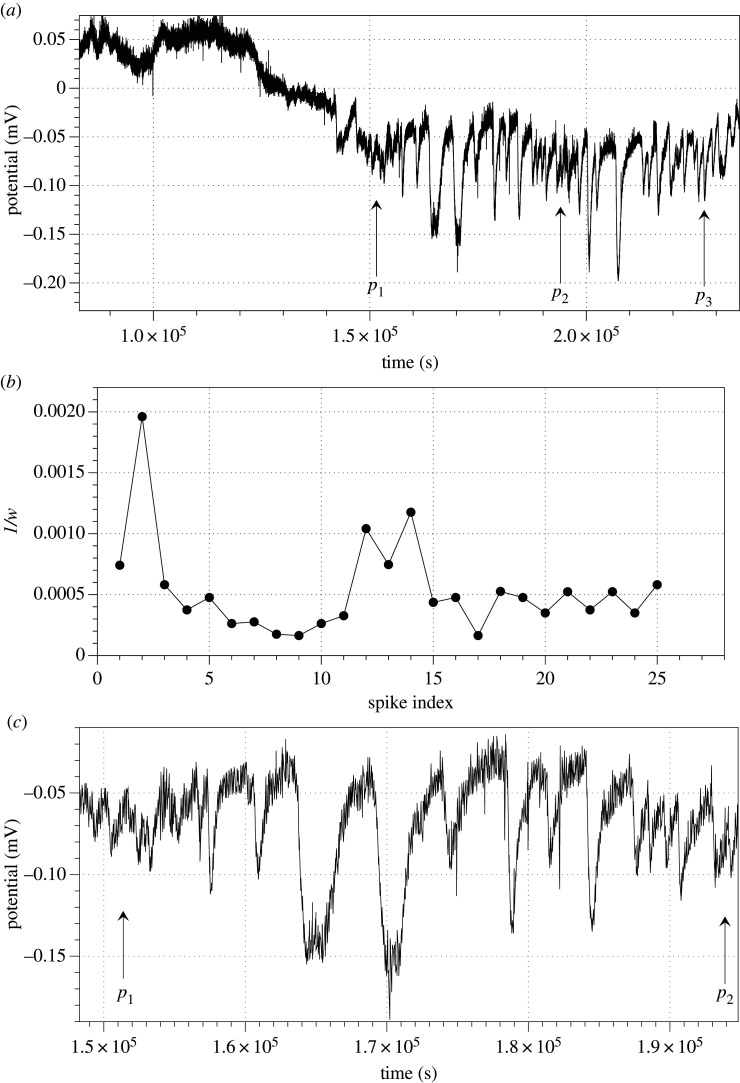
Transition to spikes outburst in *S. commune*. (*a*) There are two outbursts of spiking, first shown by arrows labelled *p*_1_ and *p*_2_ and second by *p*_2_ and *p*_3_. (*b*) Dynamical changes in frequency of spikes, as derived from (*a*). (*c*) Wave packet zoomed in, start of the packet is shown by arrow labelled *p*_1_ and end by *p*_2_.

**Figure 6.**
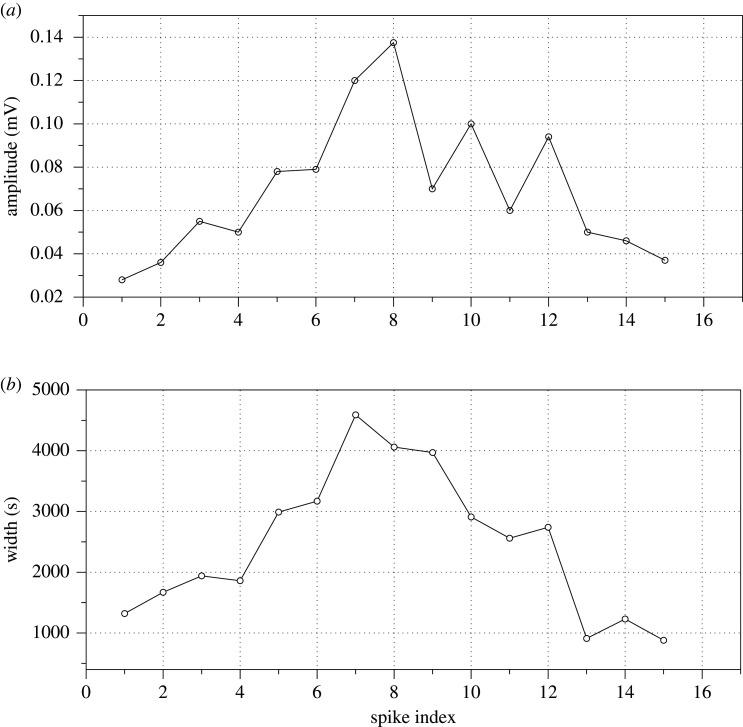
Characteristics of an exemplar wave packet of electrical potential oscillation in *S. commune*: (*a*) evolution of spike amplitude, (*b*) evolution of spike width. In a typical wave packet, spike width and amplitude increase till middle of the packet and then decrease.

*Schizophyllum commune* electrical activity is remarkably diverse (figures 3*c* and 4*e,f*). Typically, there are low amplitude spikes detected (figure 4*f*), due to the reference electrodes in each differential pair being inserted into the host wood. However, they are the fastest spiking species, with an average interval between spikes of just above half an hour (figure 4*e*). We observed transitions between different types of spiking activity from low-amplitude and very low-frequency spikes to high-amplitude high-frequency spikes (figure 5). A dynamic change in spikes frequency in the transition is shown in figure 5*b*. A closer look at the spiking discovers presence of two wave packets labelled (*p*_1_, *p*_2_) and (*p*_2_, *p*_3_) in figure 5*a*. One of the wave packets is shown in figure 5*c*, and the key characteristics are shown in figure 6.

**Figure 7.**
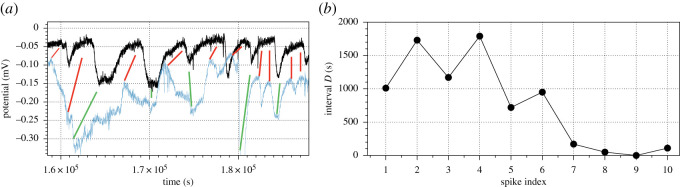
Exemplar synchronization of spikes in two neighbouring sporocarps of *S. commune*: channel (3–4), second sporocarp in figure 1*b* and channel (5–6), third sporocarp in figure 1*b*. (*a*) Spiking activity, corresponding spikes of increased voltage are linked by red lines and decreased voltage by green line. (*b*) Dynamics of the interval between spikes.

In experiments with *S. commune*, we observed synchronization of the electrical potential spikes recorded on the neighbouring fruit bodies. This is illustrated in figure 7. The dependencies between the spikes are shown by red (increase of potential spike) and green (decrease of potential spike) lines in figure 7*a*. Time intervals between peaks of the spikes occurred on neighbouring fruit bodies are illustrated in figure 7*b*. Average interval between first four spikes is 1425 s (*σ* = 393), next three spikes 870 s (*σ* = 113) and last four spikes 82 (*σ* = 73).

## 4. Towards language of fungi

Are the elaborate patterns of electrical activity used by fungi to communicate states of the mycelium and its environment and to transmit and process information in the mycelium networks? Is there a language of fungi? When interpreting fungal spiking patterns as a language, here we consider a number of linguistic phenomena as have been successfully used to decode pictish symbols revealed as a written language in [37]: (i) type of characters used to code, (ii) size of the character lexicon, (iii) grammar, (iv) syntax (word order), and (v) standardized spelling. These phenomena, apart from grammar and spelling, are analysed further.

**Figure 8.**
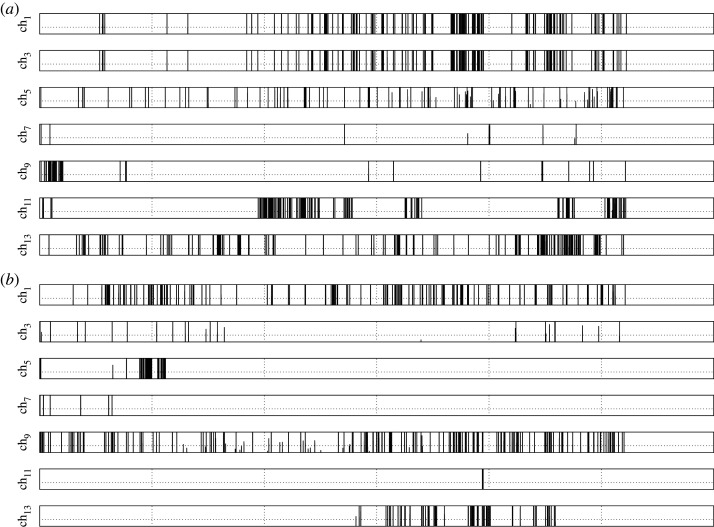
Bar-code-like presentation of spikes recorded in (*a*) *C. milataris,* (*b*) *F. velutipes*, 5 days of recording.

To quantify types of characters used and a size of lexicon, we convert the spikes detected in experimental laboratory recordings to binary strings *s*, where index *i* is the index of the sample taken at *i*th second of recording and *s*_*i*_ = 1 if there is a spike’s peak at *i*th second and *s*_0_ = 0 otherwise. Examples of the binary strings, in bar-code-like forms, extracted from the electrical activity of *C. militaris* and *F. velutipes* are shown in figure 8.

**Figure 9.**
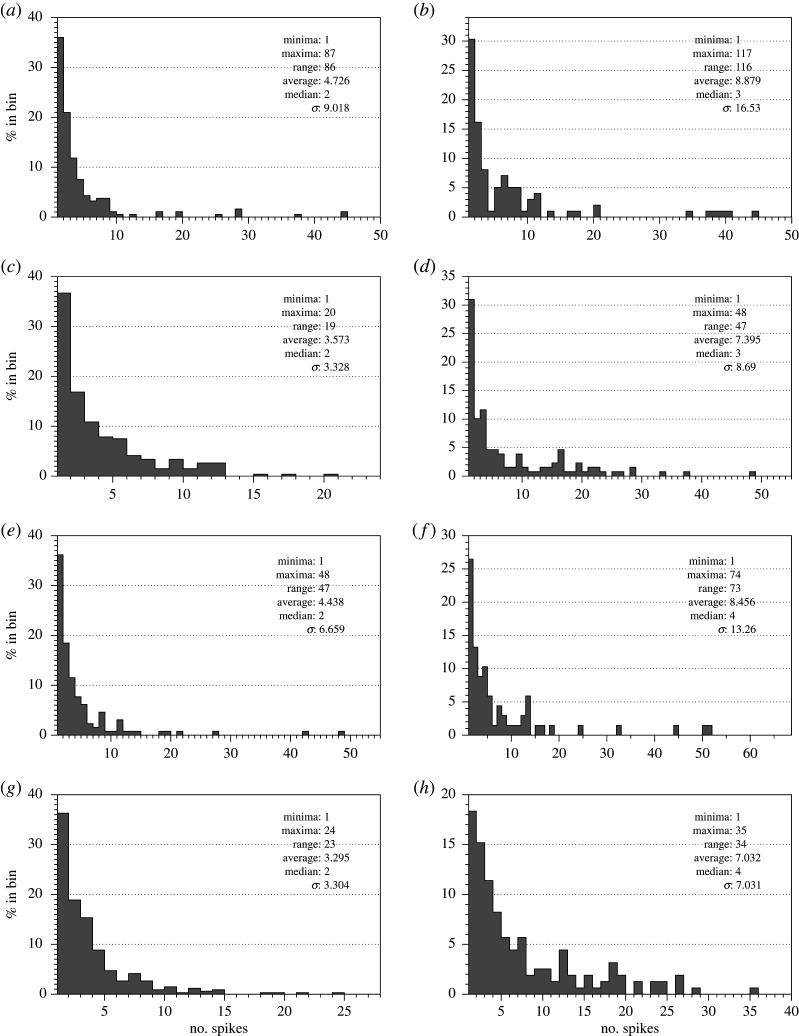
Distribution of a number of spikes in trains, i.e. of the fungal words’ lengths, of (*a,b*) *C. militaris*, (*c,d*) *F. velutipes*, (*e,f*) *S. commune*, (*g,h*) *O. nidiformis* for the train separation thresholds *a* (*a,c,e,f*) and 2 · *a* (*b,d,f,h*), where *a* is a species-specific average interval between two consequent spikes, see table 1.

**Figure 10.**
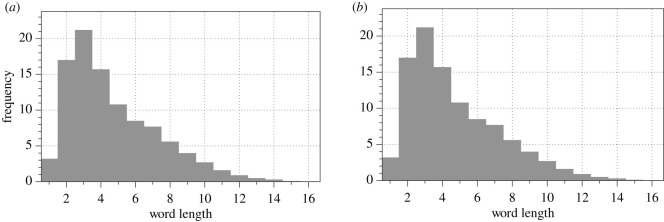
Word length frequencies in (*a*) English and (*b*) Swedish, data are taken from table 1 in [40].

To convert the binary sequences representing spikes into sentences of the speculative fungal language, we must split the strings into words. We assumed that if a distance between consequent spikes is not more than *θ* the spikes belong to the same word. To define *θ*, we adopted analogies from English language. An average vowel duration in English (albeit subject to cultural and dialect variations) is 300 ms, minimum 70 ms and maximum 400 ms [38], with average post-word onset of *ca* 300 ms [39]. We explored two options of the separation of the spike trains into words: *θ* = *a*(*s*) and *θ* = 2 · *a*(*s*), where *a*(*s*) is an average interval between two subsequent spikes recorded in species s∈{C. militaris,F. velutipes,S. commune,O. nidiformis}. Distributions of fungal word lengths, measured in a number of spikes in *θ*-separated trains of spikes are shown in figure 9. The distributions follow predictive values *f*_exp_ = *β* · 0.73 · *l*^*c*^, where *l* is a length of a word, and *a* varies from 20 to 26, and *b* varies from 0.6 to 0.8, similarly to frequencies of word lengths in English and Swedish, figure 10 and table 2 [40]. As detailed in table 2, average word length in fungi, when spikes grouped with *θ* = *a* are in the same range as average word lengths of human languages. For example, average number of spikes in train of *C. militaris* is 4.7 and average word length in English language is 4.8. Average word length of *S. commune* is 4.4 and average word length in Greek language is 4.45.

**Table 2.** Average word lengths in fungal and human languages. *l*_1_ is an average word length in the spike grouping using *θ* = *a* and *l*_2_ using *θ* = 2 · *a*, *m* is an average word length of 1950+ Russian and English language approximated from the evolutionary plots in [41] and average word length in Greek language approximated from Hellenic National Corpus [42].

	*l*_1_	*l*_2_
*C. militaris*	4.7	8.9
*F. velutipes*	3.6	7.4
*S. commune*	4.4	8.5
*O. nidiformis*	3.3	7

	*m*	
English language	4.8	
Russian language	6	
Greek language	4.45	

To uncover syntax of the fungal language, we should estimate what is most likely order of the words in fungal sentences. We do this via characterization of global transition graphs of fungal spiking machines. A fungal spiking machine is a finite state machine. It takes states from **S** ∈ **N** and updates its states according to probabilistic transitions: **S** × [0, 1] → **S**, being in a state *s*^*t*^ ∈ **S** at time *t* + 1 the automaton takes state *s*^*t*+1^ ∈ **S** with probability *p*(*s*^*t*^, *s*^*t*+1^) ∈ [0, 1]. The probabilities of the state transitions are estimated from the sentences of the fungal language.

**Figure 11.**
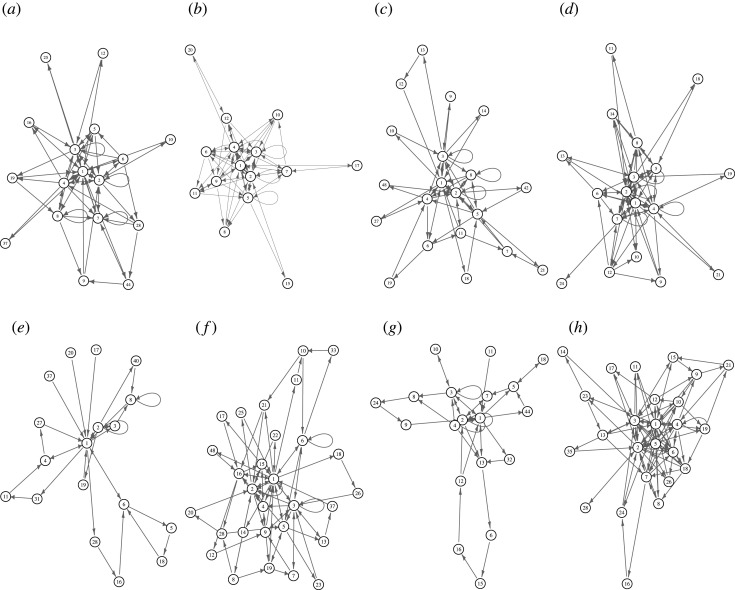
State transition graphs of fungal spiking machines, where spikes have been grouped using *θ* = *a* (*a*–*d*) and *θ* = 2 · *a* (*e*–*h*). (*a,e*) *C. militaris*, (*b,f*) *F. velutipes*, (*c,g*) *S. commune* and (*d,h*) *O. nidiformis*.

**Figure 12.**
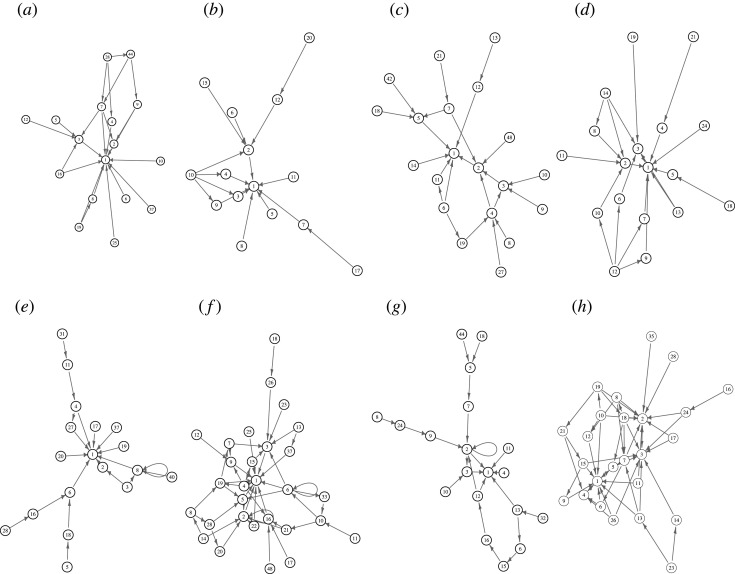
Filtered state transition graphs of fungal spiking machines, where spikes have been grouped using *θ* = *a* (*a*–*d*) and *θ* = 2 · *a* (*e*–*h*). (*a,e*) *C. militaris*, (*b,f*) *F. velutipes*, (*c,g*) *S. commune* and (*d,h*) *O. nidiformis*. The transitions were filtered in such manner that for each state *i* we select state *j* such that the weight *w*(*i*, *j*) is maximal over *w*(*i*, *z*), where *z* ∈ **S**, **S** is a set of states.

The state transition graphs of the fungal spiking machines are shown in figure 11 for full dictionary case and in figure 12 for the filtered states sets when states over 9 are removed.

The probabilistic state transition graphs shown in figure 11 are drawn using physical model spring-based Kamada–Kawai algorithm [43]. Thus we can clearly see cores of the state space as clusters of closely packed states. The cores act as attractive measures in the probabilistic state space. The attractive measures are listed in table 3. The membership of the cores well matches distribution of spike trains lengths (figure 9).

**Table 3.** Attractive cores in the probabilistic state spaces of fungal spiking machines. The attractive cores, or limit cycle, are such subgraphs of the global transition graph that when a machine enters the subgraph it will stay there forever.

	*θ* = *a*	*θ* = 2 · *a*
*C. militaris*	1, …, 8	1, …, 3,
*F. velutipes*	1, …, 8, 9	1, 2, 4, 15, 16
*S. commune*	1, …, 4, 8	1, …, 4, 7
*O. nidiformis*	1, …, 5	1, …, 5, 10, 12

A leaf, or Garden-of-Eden, state is a state which has no predecessors. *Cordyceps militaris* probabilistic fungal spiking machine has leaves ‘25’ and ‘37’ in the case of in grouping *θ* = *a* (figure 11*a*) and ‘20’, ‘17’ and ‘37’ in the case of in grouping *θ* = 2 · *a* (figure 11*e*). All other probabilistic fungal machines do not have leaves apart of *S. commune* which has one leaf ‘11’ in the case of in grouping *θ* = 2 · *a* (figure 11*g*).

An absorbing state of a finite state machine is a state in which the machine remains forever once it takes this state. All spiking fungal machines, derived in grouping *θ* = *a*, have the only absorbing state ‘1’ (figure 12*a*–*d*). They have no cycles in the state space. There are between 8 (*F. velutipes* (figure 12*c*) and *O. nidiformis* (figure 12*g*)), and 11 leaves (*S. commune* (figure 12*e*)) in the global transition graphs. A maximal length of a transient period, measured in a maximal number of transitions required to reach the absorbing state from a leaf state varies from 3 (*F. velutipes*) to 11 (*S. commune*).

State transition graphs get more complicated, as we evidence further, when grouping *θ* = 2 · *a* is used (figure 12*h*). Fungal spiking machine *O. nidiformis* has one absorbing state, ‘1’ (figure 12*h*). Fungal spiking machines *S. commune* (figure 12*g*) and *C. militaris* (figure 12*e*) have two absorbing states each, ‘1’ and ‘2’ and ‘1’ and ‘8’, respectively. The highest number of absorbing states is found in the state transition graph of the *F. velutipes* spiking machine (figure 12*f* ). They are ‘1’, ‘6’ and ‘2’. A number of leaves varies from 7, *S. commune*, to 9, *O. nidiformis* and *C. militaris*, to 12, *F. velutipes*. Only *O. nidiformis* spiking machine has cycles in each state transition graph (figure 12*h*). The cycles are 1⟷5 and 2⟷3.

To study complexity of the fungal language algorithmic complexity [44], Shannon entropy [45] and Liv-Zempel complexity [46,47] of the fungal words (sequences of spike trains lengths) are estimated using The Online Algorithmic Complexity Calculator^1^ [44,48–50] in table 4. The complexity estimates help us to rule out randomness of the electrical spiking events and to compare complexity of the fungal language with that of human. Shannon entropy of the strings recorded is not shown to be species specific, it is 2.3 for most species but 2.4 for *C. militaris* in the case of *θ* = *a* grouping and 2.5 for most species but 2.6 for *O. nidiformis* in the case of *θ* = 2 · *a*. The same can be said about second-order entropy (table 4). *Omphalotus nidiformis* shows highest values of algorithmic complexity for both cases of spike trains separation (table 4*a*,*b*) and filtered sentences (where only words with up to nine spikes are left) (table 4*c*). In order of decreasing algorithmic complexity, we then have *C. militaris*, *F. velutipes* and *S. commune*.

**Table 4.** Block decomposition method (BDM) algorithmic complexity estimation, BDM logical depth estimation, Shannon entropy, second-order entropy, LZ complexity. The measures are estimated using The Online Algorithmic Complexity Calculator (https://complexitycalculator.com/index.html) block size 12, alphabet size 256. Spike trains are extracted with (*a*) *θ* = *a* and (*b*) *θ* = 2 · *a*, where *a* is an average interval between two consequent spikes, see table 1. We also provide values of the LZ complexity and algorithmic complexity normalized by input string lengths. In table (*c*), we provide data on the strings of train powers (in number of spikes) calculated with *θ* = *a* and then filtered so values over 9 are removed and the complexity is estimated in alphabet of nine symbols.

	*C. militaris*	*F. velutipes*	*S. commune*	*O. nidiformis*
(*a*)				
algorithmic complexity, bits	1211	1052	981	1243
algorithmic complexity normalized	6.51	3.94	7.55	3.67
logical depth, steps	4321	4957	3702	5425
logical depth normalized	23	19	28	16
Shannon entropy, bits	2.4	2.3	2.3	2.3
second-order entropy, bits	3.8	3.7	3.7	3.7
LZ complexity, bits	1153	1495	910	1763
LZ complexity (normalized), bits	6.2	5.6	7	5.2
input string length	186	267	130	339

	*C. militaris*	*F. velutipes*	*S. commune*	*O. nidiformis*
(*b*)				
algorithmic complexity, bits	1047	1295	980	1393
algorithmic complexity normalized	10.57	10.04	14.4	8.82
logical depth, steps	2860	4147	3046	4731
logical depth normalized	29	32	45	30
Shannon entropy, bits	2.5	2.5	2.5	2.6
second-order entropy, bits	4	4.2	4.2	4.3
LZ complexity, bits	594	993	666	1232
LZ complexity normalized	6	7.7	9.8	7.8
input string length	99	129	68	158

	*C. militaris*	*F. velutipes*	*S. commune*	*O. nidiformis*
(*c*)				
algorithmic complexity, bits	679	976	466	1276
algorithmic complexity normalized	3.96	3.97	4.05	4
Shannon entropy, bits	2.5	2.6	2.5	2.5
second-order entropy, bits	4.7	5	4.5	4.9
LZ complexity, bits	735	1009	563	1208
LZ complexity normalized	4.3	4.1	4.9	3.8
input string length	171	246	115	319

The hierarchy of algorithmic complexity changes when we normalize the complexity values dividing them by the string lengths. For the case *θ* = *a*, the hierarchy of descending complexity will be *S. commune* (7.55), *C. militaris* (6.51), *F. velutipes* (3.94) and *O. nidiformis* (3.67) (table 4*a*). Note that in this case a normalized algorithmic complexity of *S. commune* is nearly twice higher than that of *O. nidiformis*. For the case *θ* = 2 · *a,* *S. commune* still has the highest normalized algorithmic complexity among the four species studied (table 4b). Complexities of *C. militaris* and *F. velutipes* are almost the same, and the complexity of *O. nidiformis* is the lowest. When we consider filtered sentences of fungal electrical activity, where words with over nine spikes are removed, we get nearly equal values of the algorithmic complexity, ranging from 3.96 to 4.05 (table 4c). LZ complexity hierarchy is the same for all three cases—*θ* = *a* (table 4a), *θ* = 2 · *a* (table 4b) and filtered sentences (table 4c): *S. commune*, *C. militaris*, *F. velutipes* and *O. nidiformis*. To summarize, in most conditions, *S. commune* is an uncontested champion in complexity of the sentences generated followed by *C. militaris*.

## 5. Discussion

We recorded extracellular electrical activity of four species of fungi. We found evidences of the spike trains propagating along the mycelium network. We speculated that fungal electrical activity is a manifestation of the information communicated between distant parts of the fungal colonies. We adopted a framework of information encoding into spikes in neural system [51–54] and assumed that the information in electrical communication of fungi are encoded into trains of spikes. We therefore attempted to uncover key linguistic phenomena of the proposed fungal language. We found that distributions of lengths of spike trains, measured in a number of spikes, follow the distribution of word lengths in human languages. We found that size of fungal lexicon can be up to 50 words; however, the core lexicon of most frequently used words does not exceed 15–20 words. Species *S. commune* and *O. nidiformis* have largest lexicon while species *C. militaris* and *F. velutipes* have less extensive one. Depending on the threshold of spikes grouping into words, average word length varies from 3.3 (*O. nidiformis*) to 8.9 (*C. militaris*). A fungal word length averaged over four species and two methods of spike grouping is 5.97 which is of the same range as an average word length in some human languages, e.g. 4.8 in English and 6 in Russian.

To characterize a syntax of the fungal language, we analysed state transition graphs of the probabilistic fungal spiking machines. We found that attractive measures, or communication cores, of the fungal machines are composed of the words up to 10 spikes long with longer words appearing less often.

We analysed complexity of the fungal language and found that species *S. commune* generates most complex, among four species studied, sentences. The species *C. militaris* is slightly below *S. commune* in the hierarchy of complexity and *F. velutipes* and *O. nidiformis* occupy lower levels of the hierarchy. We found that Shannon entropy poorly, if at all, discriminate between the species. That could be due to sentences in the fungal language possessing the same amount of information about physiological state of fungi and environment. LZ complexity, algorithmic complexity and logical depth give us substantial differentiation between species. The algorithmic complexity is the most ‘species-sensitive’ measure. This could be due to the fact, while conveying the same amount of information, dialects of different species are different.

Future research should go in three directions: study of inter-species variations, interpretation of a fungal grammar and reconsideration of the coding type. First, we should increase the number of fungi species studied to uncover if there is a significant variation in the language syntax among the species. Second, we should try to uncover grammatical constructions, if any, in the fungal language, and to attempt to semantically interpret syntax of the fungal sentences. Third, and probably the most important direction of future research, would to be make a thorough and detailed classification of fungal words, derived from the train of spikes. Right now, we classified the word based solely on a number of spikes in the corresponding trains. This is indeed quite a primitive classification akin to interpreting binary words only by sums of their bits and not exact configurations of 1s and 0s. That said, we should not expect quick results: we are yet to decipher language of cats and dogs despite living with them for centuries, and research into electrical communication of fungi is in its pure infant stage. And last but not least, there may be alternative interpretations of spiking electrical activity as a language. For example, one can adopt the technique of signals integration over time trace, as has been done in experiments with chemical Turing machine [55]. Another option could be to characterize each peak by determining its fuzzy entropy by the algorithm presented in [56].

## Data Availability

Data can be accessed as [57].

## Footnotes

1 https://complexitycalculator.com/index.html.
